# The Vaccine Treatment of Acne Vulgaris

**Published:** 1907-03-09

**Authors:** 


### POINTS IN TREATMENT.

### THE VACCINE TREATMENT OF ACNE VULGARIS.

NEW forms of treatment are always liable to be praised too much at first; the results of the treatment may not prove equal to expectation; the treatment is then apt to fall into disrepute. This is the danger which lies before the vaccine treatment of ordinary acne of the face or back.

Nevertheless the treatment has been followed by so much mitigation of the acne in most cases in which it has been adopted, that it thoroughly deserves extensive trial in general practice. It will probably be in the most obstinate cases that it will be first used, and in such cases relief will be welcomed even if complete cure should not follow.

The principle of the treatment is to prepare the products of the micro-organisms which cause the acne, to inject suitable quantities of these products into the tissues of the patient, with the result that the patient's tissues are stimulated to produce antibodies in larger quantities than before, and thus render the patient more resistant against the attacks of the acne organism.

The particular microbe which causes acne is the staphylococcus. There are, however, at least three varieties of staphylococci, distinguished from one another by the colour of their cultures, and accordingly termed albus, aureus, and citreus respectively. These three varieties are not always all present in the same case. Sometimes the pus from an acne spot may yield a pure culture of one variety only, sometimes two varieties may be present, sometimes all three, and in varying proportions. Moreover, the virulence of the different strains varies greatly in different patients.

In preparing the vaccine (*i.e.*, the sterilised products of growth of the cocci), it has been found that it is unsatisfactory to use one strain of staphylococci and apply the vaccine therefrom to different patients. It is very important to prepare the vaccine for a given patient from that patient's own staphylococci. This is not difficult; the fresh pus from an acne pustule is collected upon a swab or small pledget of cotton-wool, and sent to one of the many laboratories in which bacteriological work is carried on. They there smear the pus upon the surface of a suitable culture medium, incubate it, and obtain a culture of the organisms present. It is scarcely necessary to point out that no antiseptic must have been applied to the pustule, or be present in the swab or cotton-wool, or else the cocci will not grow when inoculated on to the culture medium at the laboratory. From the culture they prepare subcultures in fluid media such as broth, calculate the number of cocci per cubic millimetre of fluid, kill the cocci by heat, filter off the cocci, measure off such quantities of the fluid as contain the products of 250,000,000 staphylococci into little glass bulbs, hermetically seal the bulbs, sterilise them by heat again, and the vaccine is ready for use. The volume of fluid in each glass bulb is about 1 c.c. as a rule, and constitutes one dose. In other words, the dose is the amount of sterilised and filtered culture which contains the products of about 250 million staphylococci. The amount being only 1 c.c., it is readily given with an ordinary hypodermic needle and syringe.

In administering this dose all the usual aseptic precautions should be taken, and the vaccine injected into the subcutaneous cellular tissues just as a hypodermic injection of morphia is given; the part of the body chosen for the injection is immaterial, provided it be clean and free from pustules or spots. Probably the best site is the subcutaneous tissue of the abdominal wall.

The main objection to the use of the vaccine is naturally the necessity of giving it hypodermically. It is, however, the only beneficial way of giving it. The volume being so small, the pain is nothing more than the initial prick with the needle. Moreover, the injections have to be given only at comparatively long intervals. It has been found that the second injection is usually required about seventeen days after the first; in all, about six injections, with intervals of seventeen days between each two, may be required to produce the greatest amount of improvement; nearly always relief is already considerable after the first two.

Moreover, there have been no ill effects from the use of the carefully prepared vaccine. There is little or no feeling of illness after the injection; the patient can remain up and about doing his other ordinary work without interruption. The acne spots will begin to grow less rapidly a day or two after, the patient feeling better generally; about the seventeenth day after the first injection there will be a feeling of being less well, and the spots will begin to increase as little; then is the time for the second injection; and so on. After the cure is complete, or as complete as possible, a relapse may be met by another injection of the same vaccine; if given early the relapse should be nipped in the bud.

The use of the vaccine does not imply that the usual local treatment of the spots should be desisted from. Local treatment should be carried on at the same time. In those cases in which local treatment alone is effective in curing the acne, the vaccine treatment will naturally not be tried; it is when the local treatment is unable, by itself, to keep the acne under that vaccine treatment is likely to be of benefit.

Acne is not the only staphylococcal trouble that may be troublesome and persistent. Recurrent carbuncles, subcutaneous abscesses, some surgical and post-mortem sores, and whitlows, are all examples of possible infections by staphylococci. When recent or acute, local treatment by fomentations and so on will usually cure them at once; but sometimes the trouble is so recurrent or persistent that any additional means of treatment will be welcome. In such cases the pus should be cultivated to see whether staphylococci are the cause, in which case the vaccine may be prepared and used in precisely the same way as described above.

